# Competitive interactions between native *Spartina alterniflora* and non-native *Phragmites australis* depend on nutrient loading and temperature

**DOI:** 10.1371/journal.pone.0192234

**Published:** 2018-02-01

**Authors:** Rene Legault, Gregory P. Zogg, Steven E. Travis

**Affiliations:** Department of Biology, University of New England, Biddeford, ME; Shandong University, CHINA

## Abstract

We explored the nature and impact of competitive interactions between the salt marsh foundational plant *Spartina alterniflora* and invasive *Phragmites australis* in New England under varying levels of anthropogenic influence from nutrient loading and temperature warming. Plants were grown with and without competition in mesocosms over a four-month growing season. Mesocosms were split evenly among three levels of nutrient additions and two temperatures varying by an average of ~3° C, manipulated using small greenhouses. We measured aboveground productivity as total biomass, numbers of new stems, and mean stem height. Nutrient enrichment increased all growth parameters, while competition generally reduced aboveground biomass and the production of new stems in both species. Most importantly, smooth cordgrass suffered no negative consequences of competition when no nutrients were added and temperature was elevated. The results of this study suggest that minimizing nutrient loading into coastal marshes could be an important factor in slowing the spread of common reed into the low marsh zone of New England salt marshes as global temperatures continue to warm.

## Introduction

Anthropogenic disturbances have been shown to influence successful biological invasions of many ecosystems by non-native plant species [1]; [2]; [3]. Particularly important among these disturbances are increased temperatures due to global climate change and nutrient loading from upland runoff, which combine to alter the competitive dynamic between native and exotic species. Coastal wetlands are disproportionately affected by both nutrients, acting as “landscape sinks” for upland runoff [4], and elevated temperatures, which may increase rates of microbial decomposition resulting in elevation loss in the face of rising sea levels [5]. What is more, coastal wetlands often experience a high volume of development, and disturbance of soils is known to facilitate seed dispersal and germination among invasive plants [6]; [7]. Thus, coastal wetlands are an important arena for investigating anthropogenic influences on species invasions, notwithstanding the fact that the potential list of invaders is limited to a handful species capable of withstanding stressful conditions of high salinity and chronic hypoxia.

*Phragmites australis* (Cav.) Trin. ex Steud. (common reed) is a perennial grass that has become one of the more aggressive wetland invaders in North America [8]. The species is naturally found in temperate to sub-tropical latitudes in both the Old and the New World; however, the native lineage in North America (subs. *americanus*), has largely been supplanted by a Eurasian lineage (haplotype M; [8]) over the last half-century. This non-native lineage displays many of the aggressive intrinsic properties of other successful invasive plant species [9], including larger size [10], a rapid growth rate [11], multiple viable reproductive strategies [12], a broader tolerance to environmental stressors like salinity [13], and greater phenotypic plasticity in response to a globally changing environment [14]. The spread of non-native *P*. *australis* has become a serious management concern throughout the United States, with control efforts having little success in reducing the presence of *P*. *australis* or hindering its further expansion [6]; [15].

Human modifications to salt marsh environments, both direct and indirect, may facilitate the ability of *P*. *australis* to establish viable populations and to outcompete native foundational species. Although land use change is recognized as an important driver of *P*. *australis* expansion [16]; [17]; [18], anthropogenic changes in other factors such as temperature and nutrient loading may influence *P*. *australis* invasiveness as well. The lineage invading North America is particularly plastic to changes in temperature, and is thus expected to further expand with global warming (reviewed in [19]). Rising global temperatures increase the length of the growing season and remove some of the potential limitations to flowering in more temperate climates, possibly allowing non-native *P*. *australis* to spread more rapidly [20]. Nutrient enriched and eutrophic conditions have also been shown to give non-native *P*. *australis* an advantage over native plants [21]; [22], particularly in upland transitional and high marsh zones [23]; [24]. Such advantages have allowed non-native *P*. *australis* to consistently outperform native plant species in the high marsh zone of coastal salt marshes [25]; [26]. However, less research has been done to examine its competitive interactions with dominant low marsh plants.

In New England salt marshes, the dominant low-elevation grass, *Spartina alterniflora* Loisel (smooth cordgrass), has come under threat from the spread of non-native *P*. *australis*, given its relatively high tolerance of saline conditions [13]. Multiple studies suggest that *P*. *australis’* tendency to form dense belowground root masses at depths more than double those of native species [27]; [28]; [29] allows it to spread clonally into the low marsh where salinities might otherwise be limiting [30]; [31]. Encroachment by non-native *P*. *australis* may speed the loss of *S*. *alterniflora* marshes already under threat from a variety of anthropogenically influenced factors, including increased decomposition and coastal erosion fueled by nutrient loading [32], salt marsh die-off from herbivory [33], and sea-level rise [34]; [35].

We set out to investigate the dual effects of nutrient loading and increased temperature on competitive interactions between *P. australis* and *S. alterniflora*. We measured various aspects of productivity in both species grown in experimental mesocosms with and without competition over a four-month growing season in an attempt to shed further light on the fate of low marsh zones in the face of invasive species, anthropogenic nutrient additions, and climate disturbance. Our hypotheses were that 1) both *S. alterniflora* and *P. australis* would benefit from increased nutrient concentrations and be negatively affected by competition, 2) *P. australis* would suffer lesser negative effects of competition than *S. alterniflora* under all nutrient-enriched conditions, especially high nutrient loading, and 3) increased temperatures would slightly favor *S. alterniflora*, given its naturally broad latitudinal range and peak abundances at subtropical latitudes [36] (although note that Crosby et al. [37] found reduced survival of New England *S. alterniflora* under increased temperatures due to reductions in belowground biomass). Ultimately, this experiment was done in order to model the outcome of initial contact between native *S. alterniflora* and non-native *P. australis* to better inform conservation and management practices developed for coastal marshes of the Atlantic coast.

## Materials and methods

### Site description and plant collection

We collected *S*. *alterniflora* at Great Bay National Estuarine Research Reserve in New Hampshire (43°08’04.6”N, 70°53’13.5”W) between late May and early June, 2015. The collection site was located at the intersection of Bunker Creek and the Oyster River north of Great Bay. We sampled every 2 m along 20–30 m parallel transects running from the upland edge to the banks of tidal creeks, collecting a total of 120 sediment cores. Each core was extracted using a large “bulb planter” (6 cm diameter x 15 cm long) positioned to encompass at least five stems. We trimmed each sediment core below 15 cm to standardize core size and belowground biomass.

We collected *P*. *australis* from the Saco River estuary in southern Maine (43°28’00.4”N, 70°23’38.0”W) in early June, 2015. The collection site was dominated by non-native *P*. *australis* (confirmed by molecular marker genotyping; [38]), with the majority of stems located in high marsh conditions slightly above the average high tide mark. We used “drain spades” to extract a total of 120 single stems of *P*. *australis* along with intact roots and rhizomes. Sediment cores were trimmed to roughly the same size as *S*. *alterniflora* cores (6 x 15 cm).

A permit for the collection of plant materials from New Hampshire was provided by Great Bay National Estuarine Research Reserve. All plant materials from Maine were collected from private land with landowner permission. The Maine Department of Environmental protection considered this a de minima study and thus required no permit.

### Study design and data collection

Competitive interactions between *S*. *alterniflora* and non-native *P*. *australis* were explored by growing both species in mesocosms with and without competition, and measuring their growth across varying levels of air temperature (ambient vs. elevated) and nutrient addition (no vs. low vs. high). Mesocosms consisted of 28 cm (diam.) x 61cm (ht.) tree pots (Hummert Int., Earth City, MO) filled to a depth of 54 cm with a mixture of peat and sand (1:1 by wt). All mesocosms were divided into two halves, and plant cores were placed in the center of one side; both halves of 60 competition mesocosms received cores, whereas only one side of non-competitive mesocosms, 60 per species, received a core. Initial measurements of stem numbers and heights of both species took place immediately after planting.

Mesocosms were placed in 50-gallon plastic bins, six mesocosms to a bin, filled to 18 cm (roughly one-third of total soil depth) with half-strength (18 ppt) seawater. Salinity was maintained by keeping the volume of water constant in each bin throughout the experiment. Within each bin, two mesocosms contained only *S*. *alterniflora*, two contained only *P*. *australis*, and two contained both species. Bins, containing a total of 180 mesocosms, were organized in 3 rows of 10 separated by roughly 1 m in all directions. Each bin was randomly assigned one level of temperature and one level of nutrients. To keep the soil surface moist, all pots were watered every 1 to 2 days.

For temperature manipulation, we built a separate greenhouse for each individual bin. Greenhouses were constructed of 0.75 m (width) x 1 m (length) x 2.5 m (ht.) PVC frames wrapped in clear greenhouse plastic (Tufflite IV 006, 6mm, Berry Plastics, Evansville, IN). For the half of these greenhouses intended to produce elevated temperatures, we wrapped greenhouse plastic around the entire frame starting from the tops of the tree pots. In order to ensure similar light levels between temperature conditions, for the half of the greenhouses representing ambient temperatures, we covered three of four sides of the PVC frame in greenhouse plastic, leaving the northern side of the greenhouse open. We monitored greenhouse temperatures continuously throughout our experiment inside eight evenly spaced greenhouses using two HOBO Pendant® Temperature/Light 64K Data Loggers (Onset Computer Corp. Bourne, MA) elevated at 90 and 120 cm above the soil surface. Data loggers, which were rotated among greenhouses every two weeks, indicated a consistent temperature difference between our ambient and elevated temperature greenhouses of ~3ᵒ C during the hours of 10 AM to 4 PM each day.

For nutrient manipulation, we applied three different levels of nutrients to mesocosms: no nutrients, low nutrients (30 g N:10g P m^-2^ yr^-1^), and high nutrients (120 g N:40 g P m^-2^ yr^-1^). These levels were chosen to reflect current levels of input into New England salt marsh systems [16]; [33]. Nutrients were delivered using MiracleGro™ All Purpose Plant Food (24N-8P-16K) dissolved in deionized water. Total annual nutrient amounts were added gradually over the course of the growing season (late June to early October) in biweekly aliquots of 200 mL by hypodermic injection into the first several centimeters of soil. All mesocosms within a bin received the same nutrient treatment in order to prevent spillover effects among mesocosms.

We quantified plant performance for each species in each mesocosm at the end of our experiment in October, 2015, as total aboveground biomass, new stem production, and average stem height. Aboveground biomass was harvested in mid-to-late October by clipping all stems 2 cm above the soil surface. Plant materials were dried in an oven at 70° C to a constant mass before weighing.

### Statistical analysis

Data from each species were analyzed separately. In addition, we ran separate analyses at each of our two temperatures to assess the interactive effects of competition and nutrients on performance. Using R (Version 3.1.1, 2014), we analyzed the main and interactive effects of competition and nutrients on our three performance variables, aboveground biomass, number of new stems, and average stem height. To assure an exclusive focus on plant growth during our experiment, we took steps to factor out differences in initial plant metrics from our final measurements of aboveground biomass and average stem heights. As a surrogate for initial biomass we used stem heights summed over all stems in a mesocosm. Whether or not adjustments to our final measurements were necessary was determined by running simple linear regressions of final measurements on initial measurements and evaluating them for significant slopes. Adjusted measurements consisted of the individual residuals from these regression models. New stem production was adjusted by taking the difference between final and initial stem counts in each mesocosm. Following any necessary adjustments, we ran each analysis as a nested split-plot ANOVA with competition nested within bins nested within nutrients. Any significant main effects of nutrients or interactive effects of nutrients and competition were further explored using multiple comparisons consisting of pairwise *F*-tests (ANOVAs) after ruling out bin effects. The significance of these comparisons was assessed using Bonferroni-corrected alpha-levels (α = 0.05/3 = 0.0167 for nutrient comparisons; α = 0.05/15 = 0.0033 for competition x nutrient comparisons).

## Results

### Spartina alterniflora

**Fig 1:**
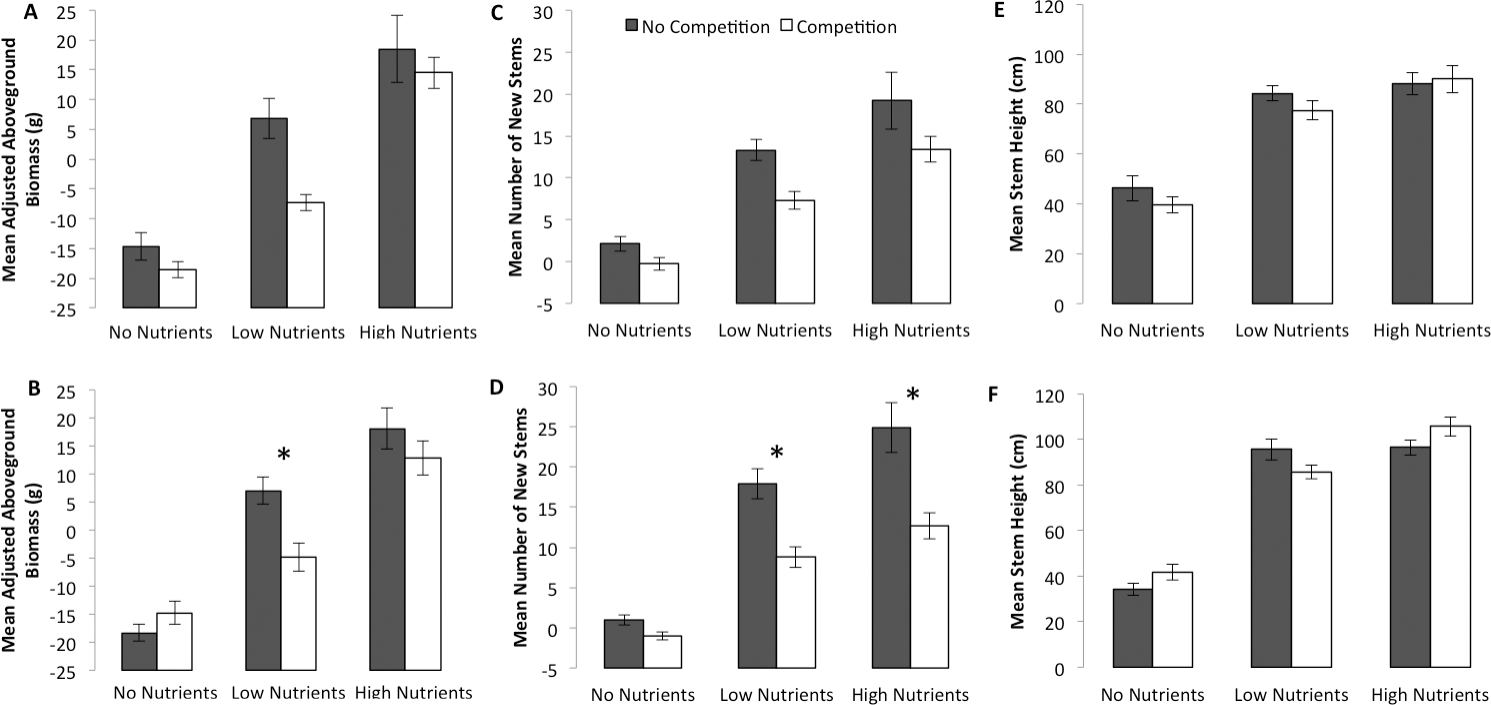
Mean (± 1 s.e.) adjusted aboveground biomass, number of new stems, and stem height of *S*. *alterniflora* at ambient (top panels) and elevated (bottom panels) temperatures. For the graphs depicting measures of productivity at elevated temperatures (bottom panels), where significant interactive effects of competition and nutrients on biomass were detected, asterisks denote significantly different mean responses between competition levels within nutrients.

Regardless of temperature, the addition of nutrients had a positive effect on the aboveground biomass of *S*. *alterniflora* (following an adjustment to factor out differences in initial biomass), whereas there was a significant main effect of competition only at elevated temperatures, which varied with nutrient level (as indicated by a significant competition by nutrient interaction, Table 1). Multiple comparisons among nutrient levels pooled over competition levels revealed that, regardless of temperature, aboveground biomass production was significantly different at each level of nutrients (F-values with 1 and 38 df ranged from 16.84 to 133.30 with all p-values <0.0005; Fig 1). At elevated temperatures, multiple comparisons among combined levels of nutrients and competition revealed that competition had no significant effect on *S*. *alterniflora* aboveground biomass at no and high nutrients, while plants grown at low nutrient levels suffered large negative effects of competition (p = 0.0028, Fig 1).

**Table 1:** F-statistics and degrees of freedom (subscript) from split-plot ANOVA models of three aboveground productivity measures taken on *S*. *alterniflora* and *P*. *australis* under ambient and elevated temperatures.

Productivity Measure	Source of Variation	Plant Species/Temperature			
Aboveground Biomass		*Spartina* ambient	*Spartina* elevated	*Phragmites* ambient	*Phragmites* elevated
	Nutrient	F_(2,12)_ = 90.28 ***	F_(2,12)_ = 41.45 ***	F_(2,12)_ = 46.45 ***	F_(2,12)_ = 42.50 ***
	Competition	F_(1,12)_ = 4.38	F_(1,12)_ = 4.91 *	F_(1,12)_ = 9.73 **	F_(1,12)_ = 6.35 *
	Nutrient x Competition	F_(2,12)_ = 0.89	F_(2,12)_ = 4.76 *	F_(2,12)_ = 0.99	F_(2,12)_ = 1.43
New Stems					
	Nutrient	F_(2,12)_ = 53.60 ***	F_(2,12)_ = 36.48 ***	F_(2,12)_ = 19.11 ***	F_(2,12)_ = 13.24 ***
	Competition	F_(1,12)_ = 11.03 **	F_(1,12)_ = 31.98 ***	F_(1,12)_ = 11.74 **	F_(1,12)_ = 25.39 ***
	Nutrient x Competition	F_(2,12)_ = 0.67	F_(2,12)_ = 4.83 *	F_(2,12)_ = 0.42	F_(2,12)_ = 0.13
Mean Stem Height					
	Nutrient	F_(2,12)_ = 40.45 ***	F_(2,12)_ = 197.15 ***	F_(2,12)_ = 22.16 ***	F_(2,12)_ = 42.40 ***
	Competition	F_(1,12)_ = 2.14	F_(1,12)_ = 0.60	F_(1,12)_ = 0.14	F_(1,12)_ = 1.76
	Nutrient x Competition	F_(2,12)_ = 1.20	F_(2,12)_ = 4.57 *	F_(2,12)_ = 1.62	F_(2,12)_ = 0.85

* = < 0.05

** = < 0.01

*** = < 0.001

In terms of numbers of new stems produced, *S*. *alterniflora* results were similar to those seen for aboveground biomass, except that the main effect of competition was significant at both ambient and elevated temperatures (Table 1). Competition accounted for 42% and 53% average overall reductions at ambient and elevated temperatures, respectively (Fig 1). At both temperatures, the addition of low nutrients significantly increased new stem production (Ambient: F_1,38_ = 56.91, p <0.0001; Elevated: F_1,38_ = 68.79, p <0.0001), whereas high nutrients further increased stem production only at ambient temperatures (Ambient: F_1,38_ = 7.34, p = 0.0100; Elevated: F_1,38_ = 4.07, p = 0.0509; Fig 1). There was another significant competition by nutrient interaction under elevated temperature conditions, where competition had no significant effect on *S*. *alterniflora* stem production when no nutrients were added, but greatly decreased stem production with either low (p = 0.0010) or high nutrients (p = 0.0029, Fig 1).

Mean stem heights (no adjustment necessary) of *S*. *alterniflora* were not significantly affected by competition, but responded positively to the addition of nutrients (Table 1, Fig 1). Under both temperature conditions, *S*. *alterniflora* produced significantly taller stems with the addition of nutrients, although the increase was almost entirely due to the addition of low nutrients (Ambient: F_1,38_ = 83.80, p <0.0001; Elevated: F_1,38_ = 194.00, p <0.0001), whereas the increase from low to high nutrients was very small in magnitude and only significant at elevated temperatures (Ambient: F_1,38_ = 3.3250, p = 0.0761; Elevated: F_1,38_ = 6.398, p = 0.0157; Fig 1). While the main effect of competition was non-significant at both temperatures, a significant interaction between competition and nutrients at elevated temperatures was detected, which was unrelated to variable competition effects within nutrient levels. Rather, the interaction was entirely due to varying effects of nutrients within competition levels. For example, low and high nutrient effects were significantly different with competition, but not in the absence of competition.

### Phragmites australis

**Fig 2:**
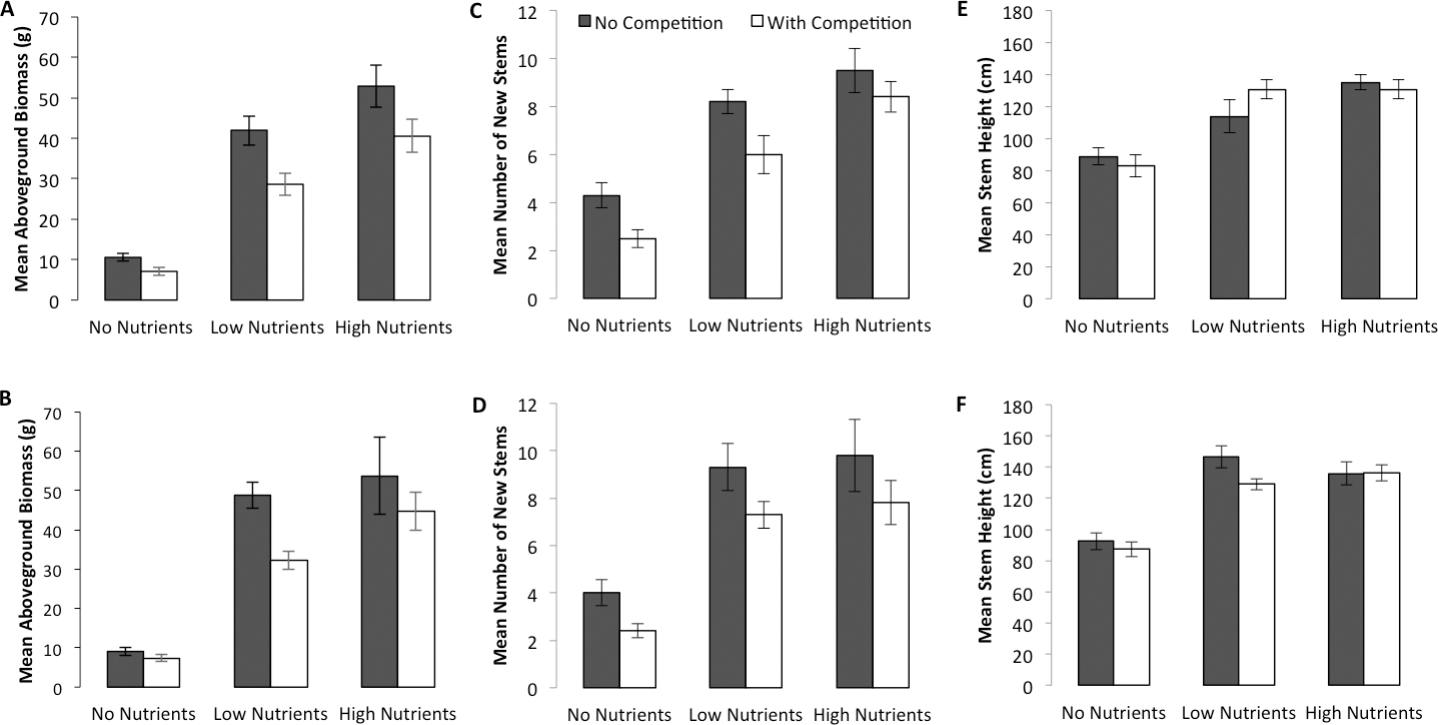
Mean (± 1 s.e.) adjusted aboveground biomass, number of new stems, and stem height of *P*. *australis* at ambient (top panels) and elevated (bottom panels) temperatures.

Regardless of temperature, competition negatively affected aboveground biomass of *P*. *australis* (no adjustment necessary), whereas the addition of nutrients had a positive effect (Table 1, Fig 2). Competition caused overall reductions in aboveground biomass across all nutrient levels of 28% and 24% on average at ambient and elevated temperatures, respectively. At both temperatures, the addition of low nutrients significantly increased aboveground biomass (Ambient: F_1,38_ = 90.22, p <0.0001; Elevated: F_1,38_ = 130.40, p <0.0001), whereas high nutrients produced further increases in biomass only at ambient temperatures (Ambient: F_1,38_ = 6.63, p = 0.0141; Elevated: F_1,38_ = 2.04, p = 0.1610).

New stem production by *P*. *australis* reflected the observed changes in aboveground biomass, with a negative effect of competition at both temperatures and a positive effect of nutrients (Table 1, Fig 2). Competition reduced new stem production by 23% and 24% on average at ambient and elevated temperatures, respectively. The addition of low nutrients more than doubled stem production at both temperatures (Ambient: F_1,38_ = 30.49, p <0.0001; Elevated: F_1,38_ = 51.64, p <0.0001), although further increases in stem production at high nutrient levels were not detected (Ambient: F_1,38_ = 5.49, p = 0.0244; Elevated: F_1,38_ = 0.21, p = 0.6500).

Nutrients, but not competition, had a significant positive effect on mean stem heights (no adjustment necessary) at both temperatures (Table 1, Fig 2). At ambient temperatures, the addition of low nutrients increased *P*. *australis* mean stem heights by 42% on average (F_1,38_ = 22.31, p <0.0001); at elevated temperatures, by 53% (F_1,38_ = 66.51, p <0.0001). The addition of high nutrients had no further effect on mean stem heights (Ambient: F_1,38_ = 2.15, p = 0.1510; Elevated: F_1,38_ = 0.88, p = 0.769).

## Discussion

The results of this study suggest that the competitive relationship between *P*. *australis* and *S*. *alterniflora* may be altered under anthropogenically disturbed conditions of increased nutrient availability and temperature. Both species showed negative responses to the effects of competition and positive responses to nutrients, mostly through changes in new stem production. More importantly, elevated nutrients failed to alleviate the negative effects of competition on *P*. *australis*, and *P*. *australis* was more negatively affected by competition with no nutrient addition than was *S*. *alterniflora*. The consistent lack of a negative competitive effect on *S*. *alterniflora* with no nutrient addition was particularly noteworthy at elevated temperatures, as reflected in a significant nutrient by competition interaction.

Several earlier studies explored the effects of competition between *S. alterniflora* and non-native *P. australis*, and our results are generally consistent, showing that competition reduces aboveground biomass in both species. For example, Peter and Burdick [39] found that competition with *S. alterniflora* significantly decreased *P. australis* aboveground biomass, shoot length, and shoot density in competitive plots. Medeiros et al. [40] found that competition between *S. alterniflora* and *P. australis* also reduced aboveground biomass, shoot height, and stem density for both species, with a significantly larger reduction for *P. australis* at higher salinities. Thus, while *P. australis* is commonly considered to be an aggressive competitor, our data support the findings of others who have shown that it is still highly susceptible to the negative effects of competition with *S. alterniflora*.

Our results indicate that changes in new stem production is the primary manifestation of aboveground competition between *P*. *australis* and *S*. *alterniflora* under anthropogenically elevated nutrient conditions. Similar greenhouse studies have found that nutrient enrichment leads to increases in stem production and aboveground biomass in *S*. *alterniflora* [41]; [42] and increases in aboveground biomass and mean stem height in *P*. *australis* [42]. Hanson et al. [41] showed increases in new stem production when *S*. *alterniflora* was enriched, although mean stem heights actually decreased. In contrast, a field study by Johnson et al. [43] showed increases in height of *S*. *alterniflora* shoots with nutrient addition, while stem density actually decreased. Differences in study design may account for this slight discrepancy: our mesocosms provided substantial bare substrate for clonal expansion, while the Johnson et al. [43] study was conducted in an established marsh.

The positive effects of nutrients on *P*. *australis* are well known from previous work [44]. Numerous other greenhouse and mesocosm studies have shown nutrients to increase biomass, new stem production, and stem height [16]; [42]; [45]; [46]. Anthropogenic nutrient loading has also been shown to promote non-native *P*. *australis* establishment and spread in the field [10]; [26]. Once established, elevated nutrients increase non-native *P*. *australis*’ aboveground biomass [47] and stem density [48], thereby threatening native salt marsh biodiversity. Nevertheless, our study suggests that *P*. *australis*’ growth and spread even in the presence of elevated nutrients is negatively affected by competition from *S*. *alterniflora* in low marsh zones.

In our experiment, the presence of a significant competition by nutrient interaction at elevated temperatures suggests an altered competitive environment for *P*. *australis* and *S*. *alterniflora* under conditions of climate warming. Previous studies have shown both *S*. *alterniflora* and multiple *P*. *australis* lineages (including invasive haplotype M) to respond to latitudinal variation in temperature in terms of biomass, stem production, and stem height, among other factors [36]; [37]; [49]. Most notably, other studies have shown increasing temperatures to increase *S*. *alterniflora* productivity aboveground by up to 45% [33]; [50] (although belowground biomass may actually decrease [37]), and to reduce stem heights and biomass in non-native *P*. *australis* [49]. In our study, *S*. *alterniflora* competed most effectively under elevated temperatures and no added nutrients. Also, aboveground biomass results at elevated temperatures differed between the two species, with *S*. *alterniflora* continuing to increase in aboveground biomass with increasing nutrient levels, while *P*. *australis* experienced no significant increase in aboveground production from low to high nutrients. One potential explanation for the competitive edge of *S*. *alterniflora* over *P*. *australis* with no added nutrients is that *S*. *alterniflora* requires fewer nutrients for organ construction [51]; [52]. The tendency of *P*. *australis* to grow taller than other salt marsh plants would presumably create an elevated demand for nutrients. Our results suggest a potential advantage for *S*. *alterniflora* in the future if nutrient loading into coastal marshes can be minimized, assuming an increase in global atmospheric temperatures of 2–3° C by 2050 [53].

While this experiment has shed further light on the competitive relationship between *S*. *alterniflora* and *P*. *australis* under anthropogenic nutrient loading and increased temperature, improvements to the study design could be made in the future. Incorporating a more realistic hydrologic regime with daily flooding would have been preferable to daily watering. This would have better simulated low marsh conditions, where waterlogging and hypoxia are more the norm, and prevented any drying out and concentration of porewater salts between bouts of watering, particularly at elevated temperatures, which is more typical of the high marsh. It would also be useful to include an additional *intraspecific* control to better account for simple density effects, with two plugs of the same species growing together in non-competition pots. Such a treatment would help to control for any limitations on nutrient and porewater availability resulting from greater stem densities irrespective of species, which may have actually exceeded pristine field conditions under our study design. Although most studies, including our own, have focused on competitive effects aboveground, Amsberry et al. [27] found that belowground interactions may actually be the primary mechanism by which *S*. *alterniflora* hinders the expansion of *P*. *australis* into the low marsh, so future studies should also include measurements of belowground biomass. Finally, simple spatial constraints inherent in our mesocosm study prevented us from simulating the effects of clonal integration on competitive interactions between the potentially very large clones characteristic of both *S*. *alterniflora* and *P*. *australis* in the field [54]; [55]. Future field experiments will be needed to adequately address this issue.

The overarching goal of our study was to investigate how nutrient additions and warming combine to influence competition between non-native *P*. *australis* and *S*. *alterniflora*, in hopes of informing and providing a framework for management of coastal marshes in New England. Our hypotheses that nutrients would increase both species’ growth and that elevated temperatures would favor *S*. *alterniflora* in competition were largely supported, though for the latter, this pattern was only consistent across productivity measures, specifically aboveground biomass and new stem production, with no added nutrients. However, the hypothesis that nutrient loading would allow *P*. *australis* to consistently outperform *S*. *alterniflora* was not supported. Based on our experimental results, predicted increases in global temperatures over the next 50–100 years have the potential to alter the competitive dynamic between these two species in East Coast salt marshes. Taking into account the data from our study, and assuming that our “no nutrient” treatments were not too far below the actual nutrient levels characteristic of pristine marshes (the 50% peat mixture we used in our experiment should have provided a relatively rich source of nutrients), it seems possible that elevated temperatures and reduced nutrient loading, in combination, could have positive effects on *S*. *alterniflora*and its ability to resist invasion by *P*. *australis*. In terms of management practices, our results suggest that managing upland runoff may be an important factor in limiting the spread of non-native *P*. *australis* and preserving native flora in salt marshes. While managers currently focus a large proportion of their efforts on removing or treating expanding patches of *P*. *australis*, we argue that preventative measures should also be implemented toward lessening the introduction of nutrients into these ecosystems.
